# Magnetic resonance imaging findings in newly diagnosed epileptic children

**DOI:** 10.12669/pjms.342.14807

**Published:** 2018

**Authors:** Mehmet Alp Dirik, Burcin Sanlidag

**Affiliations:** 1 Mehmet Alp Dirik, MD. Radiologist, Department of Radioloy, Dr. Suat Gunsel University, Faculty of Medicine, Kyrenia, North Cyprus; 2 Burcin Sanlidag, MD. Pediatrician, Department of Pediatrics Division of Pediatric Neurology, Near East University, Faculty of Medicine, Nicosia, North Cyprus

**Keywords:** Childhood, Epilepsy, Magnetic Resonance Imaging

## Abstract

**Objectives:**

Epilepsy is one of the most common chronic neurologic disorders in childhood and it affects 0.5-1% of children. The purpose of the study was to determine the prevalence and types of structural abnormalities in the epileptic children.

**Methods:**

The study was performed in Near East University and Dr. Suat Gunsel University in North Cyprus. It was conducted at pediatric neurology outpatient clinic of the hospital. The records of 1 to 18 years old epileptic children in whom Magnetic Resonance Imaging (MRI) performed within 6 months after diagnosis were enrolled to the study between the dates of October 2011 and June 2017.

**Results:**

Among 220 children; 131 (59.55%) had no abnormality and 89 (45.45%) had at least one abnormality in the MRI. Most commonly documented lesions were generally encephalomalacia, hydrocephaly and brain atrophy with a percent of 5.90 (13 cases), 5.45 (12 cases) and 4.55 (10 cases) respectively. Sixty nine (31.06%) of the patients had one abnormality whereas 20 (9.09%) had two or more lesion.

**Conclusion:**

Abnormality in MRI examination in newly diagnosed epileptic children was high. These high rates may be due to enrollment of children with new emerging epilepsy on a chronical neurologic disorder. Additionally 20 (9.09%) of patients had a concomitant lesion. Secondary lesions were detected in cases with corpus callosum abnormality, atrophy, encephalomalacia and hydrocephaly. Primarily formed lesions are unknown; further studies are needed to confirm these findings.

## INTRODUCTION

Epilepsy is one of the most frequently seen neurologic disorders within childhood. International League against Epilepsy defines epilepsy as; at least two unprovoked or reflex seizures >24 h apart. Epilepsy affects 50 million people around the world and half of them start in childhood period.^1^-^3^

King et al. reported abnormal Magnetic Resonance Imaging (MRI) findings when all seizures evaluated as 35 in 263 patients (13.3%).^4^ With the increase of availability of high quality MRI; lesions that are not detected in Computed Tomography such as heterotopias and mesial temporal sclerosis that are both associated with childhood onset seizures can be visualised.^5^-^8^ Superiority of MRI to CT is especially in temporal lobes because; inferior temporal lobes may be inapperent cause of beam hardening artefacts on CT.^9^ Particularly high quality MRI has increased the understanding of underlying mechanisms. Thus; evaluation and management of epilepsy had also be changed.^10^

Lack of radiation is another superiority of MRI according to CT.^11^ With emerging new modalities; neuroimaging becomes more important in the improvement of epilepsy. ILEA has recommended neuroimaging with an MRI for all patients with epilepsy.^5^ Therefore; neuroimaging in childhood epilepsy is unavoidable for the determination of possible etiological cause, prognosis and therapeutic approaches. First choice of imaging modality is MRI with special protocols for epilepsy.

Most published imaging studies of pediatric age are about first seizure targeting to clarify reasons of acute symptomatic seizures like meningitis, encephalitis, trauma, Central Nervous System (CNS) hemorrhage in order to plan immediate tratment.^12^-^14^

In a recent study of pediatric brain imaging in epilepsy 55.86% of patients showed abnormal MRI findings. Mild generalized brain atrophy was demonstrated as 12.91% being the leading abnormality.^15^ Aim of the present study was to find out the prevalence and types of structural abnormalities in the children who has newly diagnosed as epilepsy.

## METHODS

This was a retrospective study. The records of one to 18 years old epileptic children who admitted to pediatric neurology outpatient clinic between October 2011 and June 2017 were evaluated. Two hundred and twenty children who were examined with MRI within six months of diagnosis were enrolled in the study.

The MRI examination was performed by using a 1.5T (Siemens) and 3T (Siemens) devices with a pediatric epilepsy protocol. The standardized protocols consist of the following scanning sequences: sagittal T1-weighted spin echo, axial T2-weighted fast spin echo, coronal oblique fast fluid-attenuated inversion recovery, coronal oblique fast multiplanar inversion recovery, axial diffusion (single-shot, spin echo planar), b = 1000, all directions), and axial three-dimensional spoiled gradient recalled echo.

The total imaging time for all sequences is about 26 minutes. A subset of 34 examinations was performed on MRI units at referring facilities. These were performed on magnet systems varying from 0.5 to 1.5 Tesla and consist of standard sagittal, axial, and, on most patients, coronal images, using typical clinical sequences. All these studies were rated as adequate or better in scan quality. Gadolinium was administered as MRI contrast.

### Statistical Analysis

The data was analyzed by SPSS-19. The results were described and compared.

## RESULTS

A total of 220 children with the diagnosis of epilepsy underwent MRI examination within six months of diagnosis; of whom 131 (59.55%) had no abnormality detected by the MRI. Among 220 children; 89 (40.45%) demonstrated abnormality on the MRI study of the brain. Sixty nine (31.36%) of children had one characteristic abnormality, whereas 20 of the children (9.09%) had 2 or more lesions in the neuroimaging.

Findings were categorized as focal and non-focal lesions. Focal lesions detected in 30 (13.64%), and non-focal lesions demonstrated in 82 (37.27%) of the patients. Abnormal findings are summarized in Table-I.

**Table-I:** Abnormal Brain MRI findings.

MRI findings	Number of patients among ones with positive MRI finding (n)	Percentage (%)
**Focal lesions**	**30**	**13.64**
Tumor	2	0.90
Cortical tuber	1	0.45
Mesial temporal sclerosis	1	0.45
Porencephalic cyst	3	1.36
Venous angiom	3	1.36
Hypocamppal sclerosis	2	0.90
Cysts	7	3.18
-neuroglial cysts	3	1.36
-pineal cyst	1	0.45
-arachnoid cyst	3	1.36
Other focal lesions	8	3.37
-focal ischemic lesion	2	0.90
-focal gliosis	1	0.45
-previous hemorrhage	5	2.27
Focal encephalomalacia	3	1.36
**Non- focal lesions**	**82**	**37.27**
Axonal injury	1	0.45
Encephalomalacia	13	5.90
Hydrocephaly	12	5.45
-without shunt	11	5
-with shunt	1	0.45
Hypomyelination	5	2.27
Diffuse atrophy	10	4.55
Corpus callosum pathology	9	4.09
-hypoplasia	4	1.82
-dysgenesis	3	1.36
-agenesis	2	0.90
Cerebellar atrophy	5	2.27
-with cerebral atrophy	3	1.36
-without cerebral atrophy	2	0.90
Heterotrophy (pachygyria, polymicrogyria)	5	2.27
Periventricular leukomalacia	5	2.27
Leukomalacia	3	1.36
Gliosis	7	3.18
Mega cisterna magna	1	0.45
Increased signal intensity	6	2.72

The most commonly demonstrated pathology was encephalomalacia with a percent of 5.90 (n: 13). Focal encephalomalacia was reported in 1.36 % (n: 3) of patients. The second most common abnormality was hydrocephaly encountering 5.45% (n: 12). Generalized atrophy demonstrated in 10 of the patients constituting 4.55% of the study population. Five (2.27%) of the patients had cerebellar atrophy. Within cerebellar atrophy group of patients; three of them had accompanying cerebral atrophy; whereas two of them were isolated cerebellar atrophy. Corpus callosum abnormalities detected in 4.09 % (n: 9) of the patients. Generalized gliosis demonstrated in seven patients being 3.18% of the population. Periventricular leukomalacia were recorded in five (2.27%) patients. Leukodystrophy noted in three patients (1.36%).

Among focal lesions; the prevalence were determined as of tumor 0.9% (n:2), porencephalic cyst 1.36% (n:3), venous angioma 1.36% (n:3) and hippocampal sclerosis 0.9% (n:2) respectively.

Cysts was found in 7 (3.18%) patients of which; three of them were cortical neuroglial, three of them were arachnoid and one of them was pineal cyst. Heterotropy detected in 5 (2.27%) of the patients. Cortical tuber, axonal injury, mega cisterna magna; each abnormality detected in a single patient of the study group being 0.45% each.

Non-specific increased signal intensity in the white matter was found in 2.72 % (n: 6) of the patients. Twenty patients had two or more lesions and accompanying lesions were noted in cases with corpus callosum abnormality, cerebral atrophy, encephalomalacia and hydrocephaly. Prevalence of a secondary lesion in case of corpus callosum abnormality, cerebral atrophy, encephalomalacia and hydrocephaly were 77.78%, 60%, 50% and 50% respectively. Types of concomitant lesions were summarized in Table-II.

**Table-II:** Types of lesions accompanying corpus callosum abnormality, cerebral atrophy, generalised encephalomalacia and hydrocephaly.

Corpus callosum abnormality	Cerebral atrophy (6/10; 60%)

Number of cases with secondary abnormality /Number of total cases: 7/9 (77.78%)	Number of cases with secondary abnormality/ Number of total cases: 6/10 (60%)
Encephalomalacia	Cerebellar atrophy
Hydrocephaly	Hypoplasia of corpus callosum
Previous hemorrhage	Hydrocephaly
Cerebral atrophy Hypomyelination	Hippocampal sclerosis

*Generalised encephalomalacia*	*Hydrocephaly*

Number of cases with secondary abnormality / Number of total cases: 8/13 (61.53%)	Number of cases with secondary abnormality/ Number of total cases: 6/12 (50%)
Hydrocephaly	Cerebral atrophy
Gliosis	Porencephalic cyst
Mega cisterna magna	Arachnoid cyst Heterotrophy (pachygyria)

## DISCUSSION

Cumulative incidence of epilepsy is 3 % all over the life and more than half of them are started since childhood period.^2^ Epilepsy affects 0.5-1% of children and it is one of the most common chronic neurologic disorder among children. Incidence is higher in underdeveloped countries, when compared with developed countries.^16^

Neuroimaging in the early course of epilepsy is important as it can identify etiology and guide therapeutic approaches. MRI imaging can identify nonspecific abnormalities like periventricular leucomalacia, atrophy, it can demonstrate static remote lesions like porencephaly, it can point out focal lesions responsible for seizures like focal cortical dysplasias and mesial temporal sclerosis being a potential candidate for epilepsy surgery. Also it is useful for the diagnosis of subacute and chronic processes like metabolic disorders and it is important for identifying acute process requiring emergent intervention like tumor, stroke, encephalitis and hydrocephalus.^17^

In the conducted study, 40.45% of the children who were diagnosed with epilepsy had an abnormal MRI finding. In different studies evaluating brain imaging findings in childhood epilepsy; abnormal MRI findings varies between 28.5-55.86%.^2^,^15^,^18^ Our result is in between those reports. Betting et al demonstrated abnormal MRI findings in 24% of patients only with idiopathic generalized seizures.^19^ However in our study all epileptic children were enrolled which may be the reason of higher rates of abnormality may be because of that.

Due to different patient groups, comparison of results between the conducted study and the others is quite difficult. Encephalomalacia was the leading abnormal finding our study. Among encephalomalacias; 13 were generalized and three of them were focal; constituting a percent of 7.27 for overall study population. Kalnin and colleagues documented encephalomacia as 6% in children being evaluated after first seizure.^13^ As an opposing view to our study; Amirsalari et al. reported abnormalities in epileptic children including brain atrophy, benign cycts, vascular abnormalities, brain tumors and increased white matter signal intensity.^2^

Epilepsy and hydrocephaly are well known to occur together. In a recent study enrolling 411 infancy-onset hydrocephalus; 18 % showed to have epilepsy.^20^ Hydrocephaly was the second most common abnormal finding with a percentage of 5.45 in this study. In another study in epileptic children; hydrocephaly was documented as 3.82%.^15^ Ventricular enlargements was reported by Kalnin et al. as the leading abnormality in children with first recognized seizures. In that study; among mild ventricular enlargement group 7 of 39 and among moderate ventricular enlargement group all five children had other significant accompanying abnormalities.^13^ Within our study group half of the cases had abnormality like cerebral atrophy, porencephalic cyst, arachnoid cyst or pachygyria (Table-I).

The brain atrophy was detected as 3rd common abnormality being 4.55%; which is lower than the ratios reported by Amirsalari et al. as 10% and Ali et al. as 12.91%.^2^,^15^ Brain atrophy was the most commonly reported abnormality in those studies unlike our findings. This difference may be due to performing of MRI within just after or within a short time interval after diagnosis of epilepsy.

The most common cause of epilepsy in developed countries were cerebral dysgenesis followed by hypoxic-ischemic lesions, metabolic disorders and tumors.^21^,^22^ In the present study those were reported with different proportions as the study performed in a different population (Table-I).

There is an increasing evidence of cerebellar atrophy in adults with epilepsy. In new onset epilepsy group in adults and after first seizure evaluation of children; no cerebellar atrophy had been documented.^13^,^23^,^24^ It was postulated to be correlated with duration of the disorder and number of lifetime seizures.^13^ In our study the cerebellar atrophy was detected in 5 (2.27%) of the patients; in which three of them were together with cerebral atrophy and two of them were alone. This may be due to new onset of seizures within the course of a chronical neurologic disease like inborn hypoxia. In another study the rate of cerebellar atrophy in epileptic children was documented to be 1.43%.

The number of children with two or more lesions in MRI was 20 (9.09 %). Similarly in a study evaluating MRI abnormalities in children after first recognized seizure; they found in more than one lesion in 12% (34/281).^13^ Corpus callosum abnormality (7 of 9 patients), cerebral atrophy (6 of 10 patients), generalized encephalomalacia (8 of 13 patients) and hydrocephaly (6 of 12 patients) were found to have accompanying abnormalities. Which abnormality came first is unknown.

Another important point is that existence of an abnormal finding in MRI had been reported to be related with prognosis of the disease. Gaillard at al reported that in partial seizures, outcome can be predicted with initial MRI results.^25^ Similarly in temporal lobe epilepsies MRI abnormalities had been found to be only independent predictor of seizure outcome.^26^ So, neuroimaging findings are important in the setting of therapeutic approaches in childhood and to detect evaluation of the disease to adulthood period.

## CONCLUSION

Abnormality in MRI examinations was noted in 45, 45% of patients with newly diagnosed epilepsy. Neuroimaging especially with high resolution MRI integrated to workflow in childhood epilepsy in understanding underlying pathophysiology, to assist treatment planning and to estimate prognostic outcome. Twenty (9.09%) of the cases had more than one lesion on MRI. Secondary lesions were observed in cases with corpus callosum abnormality, atrophy, encephalomalacia and hydrocephaly. Which lesions had formed primarily is unknown which warrants further investigation. Possible new modalities of MRI will make these issues more certain and outcomes will be more predictable.

Important findings of the study were that approximately half of the children with newly diagnosed epilepsy had abnormal findings in MRI. Additionally; 9.09% of the study population had more than one lesion on MRI. Encephalomalacia was the leading abnormal finding in this study. Concomittant lesions were detected in cases with corpus callosum abnormality, atrophy, encephalomalacia and hydrocephaly. However primarily formed lesions are unknown requiring further investigation.
